# Supplementary figures and images for: Adapting to altered auditory cues: Generalization from manual reaching to head pointing
Source: PLoS One. 2022 Apr 14;17(4):e0263509. doi: 10.1371/journal.pone.0263509 (PMC9009652; doi:10.1371/journal.pone.0263509)

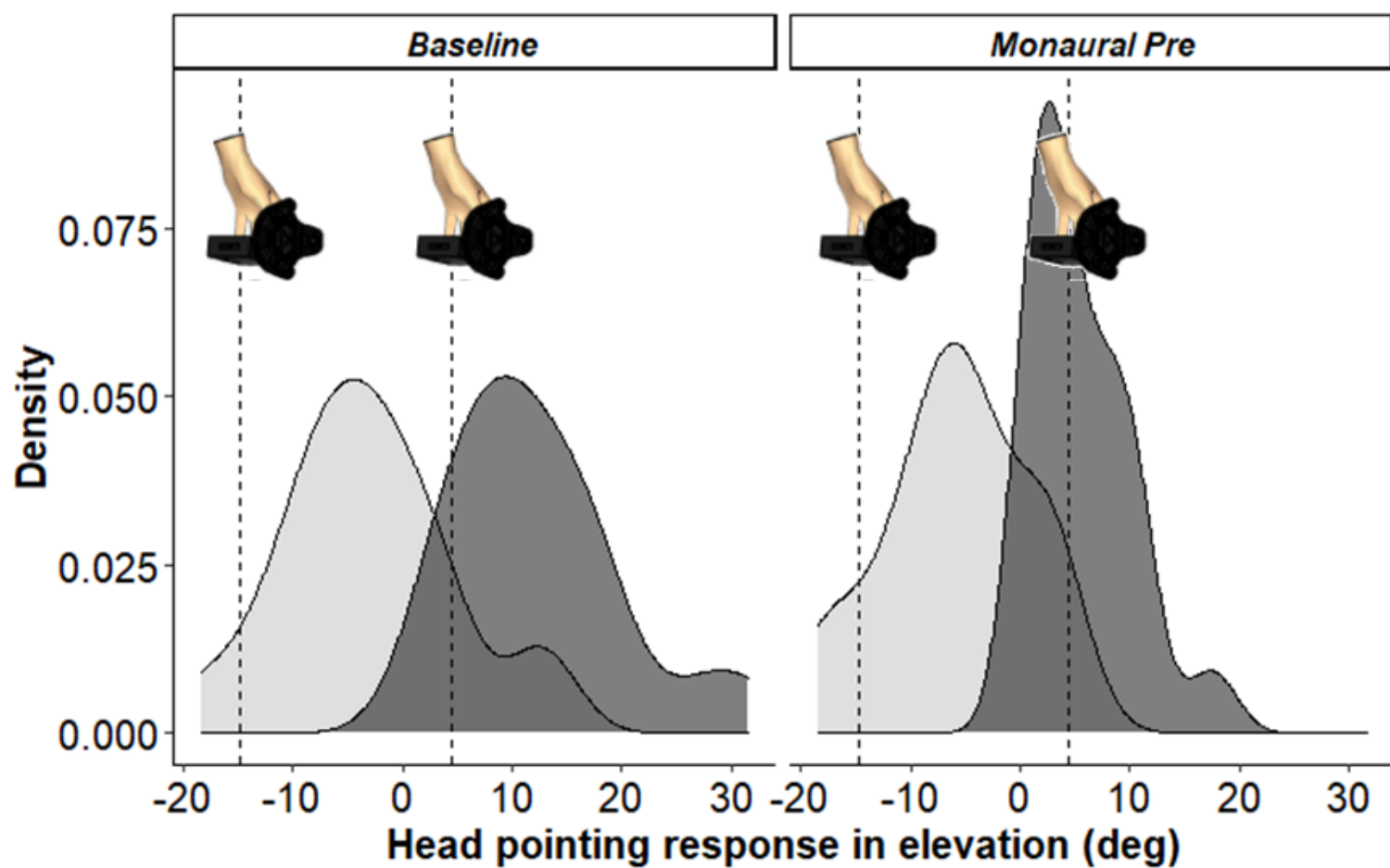

Supplement: S1 Fig — Density plot of responses during Session 1, as a function of sound position in elevation. The center of the speaker was located at -15 or +5 with respect to the ear level (shown by dashed lines), depending on the trial. This asymmetrical arrangement of the speakers resulted from the fact that the software for placing the speakers at pre-determined positions was based on the position of the VIVE tracker above the speaker, rather than the position of the actual speaker. (PDF) [file pone.0263509.s001.pdf]
