# Supplementary material for: Adapting to altered auditory cues: Generalization from manual reaching to head pointing
Source: PLoS One. 2022 Apr 14;17(4):e0263509. doi: 10.1371/journal.pone.0263509 (PMC9009652; doi:10.1371/journal.pone.0263509)
Supplement: S1 Table — LME analyses of absolute and signed errors in azimuth and elevation. (PDF) [file pone.0263509.s002.pdf]

	X^2	*df*	*p*
**AZIMUTH Absolute error**			
TESTING PHASE	76.45	1	<.001
TRAINING TYPE	0.14	1	.071
AZIMUTH SOUND POSITION	20.09	1	<.001
TESTING PHASE* TRAINING TYPE	19.18	1	<.001
TESTING PHASE* AZIMUTH SOUND POSITION	7.78	1	.005
TRAINING TYPE * AZIMUTH SOUND POSITION	5.39	1	.02
TESTING PHASE* TRAINING TYPE * AZIMUTH SOUND POSITION	3.39	1	.07
**AZIMUTH Signed error**			
TESTING PHASE	58.07	1	<.001
TRAINING TYPE	0.05	1	.082
AZIMUTH SOUND POSITION	9.96	1	.002
TESTING PHASE* TRAINING TYPE	27.26	1	<.001
TESTING PHASE* AZIMUTH SOUND POSITION	0.21	1	.65
TRAINING TYPE * AZIMUTH SOUND POSITION	1.53	1	.22
TESTING PHASE* TRAINING TYPE * AZIMUTH SOUND POSITION	7.18	1	.007

**ELEVATION Absolute error**			
TESTING PHASE	3.40	1	0.07
TRAINING TYPE	3.78	1	0.05
TESTING PHASE* TRAINING TYPE	0.46	1	0.50
**ELEVATION Signed error**			
TESTING PHASE	22.31	1	<.001
TRAINING TYPE	12.67	1	<.001
TESTING PHASE* TRAINING TYPE	2.68	1	0.10
