# Supplementary material for: Adapting to altered auditory cues: Generalization from manual reaching to head pointing
Source: PLoS One. 2022 Apr 14;17(4):e0263509. doi: 10.1371/journal.pone.0263509 (PMC9009652; doi:10.1371/journal.pone.0263509)
Supplement: S2 Table — Mean ± SD for absolute and signed errors in azimuth as a function of 8 target positions and PHASE (pre or post training) and training (Spatial or Non-Spatial during monaural listening). (PDF) [file pone.0263509.s003.pdf]

			-15				5			
	PHASE	TRAINING	**-67.5**	**-22.5**	**22.5**	**67.5**	**-67.5**	**-22.5**	**22.5**	**67.5**
**Absolute error**	Baseline		5.9±8.5	3.0±1.8	2.9±1.2	6.2±10.3	5.6±9.7	3.1±1.3	3.0±1.9	5.1±8.4
	Pre	Spatial	12.0±12.3	18.5±11.6	22.1±14.8	23.6±15.7	14.5±13.9	16.5±11.8	23.6±16.8	23.5±15.4
	Post	Spatial	10.9±9.5	9.9±7.9	10.4±10.8	13.5±10.9	9.5±9.2	10.8±9.2	13.2±12.0	13.6±9.4
	Pre	Non-Spatial	12.4±8.1	17.8±9.9	24.1±15.4	25.5±21.9	10.9±9.0	16.1±8.6	20.1±14.8	26.3±20.3
	Post	Non-Spatial	9.7±6.2	12.6±9.1	14.6±12.3	21.5±17.7	10.5± 7.4	12.5±9.3	14.4±11.6	20.5±16.9
**Signed error**	Baseline		1.5±10.0	-0.8±3.0	0.5±2.6	-3.1±11.5	1.5±10.9	-0.3±2.8	0.2±2.8	-1.2±9.6
	Pre	Spatial	-7.0±15.5	-16.9±13.5	-15.5±21.9	-21.3±18.9	-7.9±18.4	-15.9±12.5	-14.9±25.1	-21.2±18.5
	Post	Spatial	-2.3±13.4	-3.7±11.0	-3.6±14.2	-7.5±15.5	-1.8±12.5	-9.1±10.3	-5.2±16.4	-8.2±13.7
	Pre	Non-Spatial	-8.9±11.6	-15.0±13.2	-17.7±22.1	-23.2±24.4	-7.2±11.7	-13.0±13.0	-13.5±21.1	-23.7±22.7
	Post	Non-Spatial	-4.0±9.8	-8.2±12.6	-9.7±16.2	-19.3±19.8	-3.6±12.0	-10.8±10.5	-7.2±16.7	-19.1±18.3
