# Supplementary material for: Adapting to altered auditory cues: Generalization from manual reaching to head pointing
Source: PLoS One. 2022 Apr 14;17(4):e0263509. doi: 10.1371/journal.pone.0263509 (PMC9009652; doi:10.1371/journal.pone.0263509)
Supplement: Attachment — Submitted filename: Response.docx [file pone.0263509.s004.docx]

**Reviewer #1**

This study examined the effect of spatial and non-spatial auditory training on the localisation of sounds and on auditory cueing of visual stimuli. Spatial training improved spatial localisation, but not spatial cueing. Lower improvements were seen for non-spatial training. Importantly, the authors report that spatial training with arm reaching generalised to localisation with movements of the head, suggesting integration of auditory, arm and head coordinate frames. However, it is not clear to what extent participants moved their head, as well as their arms, during training. Conclusions about generalisation are therefore difficult to draw.

We thank the Reviewer for raising the issue of head-movements during training and during testing and inviting us to elaborate further on this aspect. In the Discussion we have now added the following paragraph to address this topic:

“One important aspect to notice is that head movements during the Spatial training were not only functional to orient the head to the target selected for reaching. Actually, most head-movements preceded reaching, hence they were spontaneously implemented by participants. Although 61.5% of trials fell within 10 degrees from the speaker reached with the hand, in the remaining proportion of trials the head was not aligned with the hand. In fact, the misalignment between the hand and the head was 9.7 degrees (SD = 8.7) when the hand reached the target speaker. Moreover, during the Spatial training, participants made 2.2 movements (on average) in each trial, and the timing of head-movements and reaching actions differed. The first head movement during the Spatial training started around 1 second after the beginning of the sound, whereas the first hand-contact with the speaker in each trial occurred around 4 seconds after the beginning of the sound. Most notably, the rightward bias of the head we described above indicates that head movements were not only performed to coordinate head-pointing with the hand-reaching response. Taken together these findings indicate that participants explored the auditory scene with their head before taking the decision to act on the sound source. One important implication of this finding is that head-movements during the Spatial training likely served two purposes: exploration of the acoustic scene and support for the reaching response phase. The latter coupling between head-movements and reaching response reveals a partial overlap between the motor response required for the untrained task (head-pointing to sound task) and the Spatial training task (head movements that accompanied the hand reaching response). Future work should address generalization also using auditory tasks that do not entail any such overall in the motor response between trained and untrained task (e.g., assessing generalization through minimum audible-angle tasks, as in 13).”

Also, the authors point to the significant effect of spatial training on localisation, but not on cueing. The statistics for cueing are only cursorily described and the data are relegated to the Supplementary Material. To what extend are these results important to the conclusions of the study? They could be more visible and more thoroughly discussed.

We agree and we have now moved the figure about the AV attention cueing results from the supplementary materials to the main text (now Figure 8). In addition, we now provide more details about these results and we examine them in a dedicated paragraph in the Discussion (see page 29).

Specific comments

Head-pointing localisation task: While it is true that head movements made during sound presentation will dynamically change auditory cues to location, thereby reducing ambiguity, head-pointing to sounds is a special case where interaural cues can be nulled. The ear plugged conditions are only monaural to the extent that the plug/muff combination sufficiently attenuates sound. To what extent would interaural time differences still be available at low frequencies where plugs/muffs are less attenuating? Could it be that participants are able to null residual ITDs?

One clear finding of our study is that participants moved their head to expose their unplugged left ear to the sounds, irrespective of whether they occurred on the left or right side. This was evident both during the Spatial training and in the immediate head-movements performed in the head-pointing to sound task after the Spatial training. In both cases a clear rightward bias in head-pointing emerged.

The Reviewer’s hypothesis that participants solved the task by nullifying binaural cues is not compatible with this rightward bias. If participants had exploited any residual ITDs at low frequencies, we should have observed correct head-movements to the sounds (resulting in no lateralised bias, when averaging responses across all sound positions). Instead, if participants had exploited the unbalanced ILDs to nullify the interaural difference, they should have shown a leftward bias (not a rightward one). This because they would have need to expose the plugged ear more to the sounds in order to nullify the level difference in the context of the plug.

We nonetheless improved our discussion of the relation between head-movements and auditory cues thanks to this prompt from the Reviewer. The changes are now introduced at page 28-29.

Line 74. Head movements and localisation accuracy were both higher in the reaching group than in the naming group. Can you be sure that it was arm reaching and not head movements that caused the improvement in localisation?

We indeed think that arm reaching may promote adaptation to altered auditory cues by favouring the development of head-movements. We have now clarified this point in the Introduction (see pages 4-5).

Figure 2 is duplicated as Figure 3. Figure 3 is missing.

We apologize with the reviewer for this mistake. We have now added the correct Figure 3.

As I understand it, the linear mixed effect model estimates the linear regression of error on azimuth, with listening condition and participant as fixed and random factors. Is there any reason to assume that the relationship of error and azimuth is linear? The rationale for examining the linear trend across azimuth could be better articulated.

We based our decision to model the data distribution as linear on visual inspection of the average data. As shown in the figure below, the more sound position is close to the plugged space (right space, indicated by positive values on the x-axis), the more absolute error increases. This increase appears to be best approximated by a linear trend. This is also in line with previous findings, that also observed a similar trend in the data and hence adopted linear models (see: Valzolgher, C., Campus, C., Rabini, G., Gori, M., & Pavani, F. (2020). Updating spatial hearing abilities through multisensory and motor cues. Cognition, 204, 104409).

The main reason why we did not report average data in Figure 2, 3 and 4 (and showed only the model fitting) was to avoid cluttering and to improve figure readability. Should the Reviewer prefer to see the average data plotted on the figure we could surely add them.

Figure 2C: Can you speculate why localisation bias changed across source azimuth in the monaural condition? For each location, the head would have been more-or-less centred on the source at time of response. If localisation cues were head-centred, one would have expected similar results across source locations.

This bias refers to the fact that when one ear is plugged, listeners perceive sounds ipsilateral to that ear as attenuated. Hence, when prioritising binaural cues (ILD in particular) they misinterpret the interaural difference and perceive the sound source toward the open ear. As the distance from the open ear increases, this bias also increases. This type of observation has been made in previous studies. See for example:

- Valzolgher, C., Campus, C., Rabini, G., Gori, M., & Pavani, F. (2020). Updating spatial hearing abilities through multisensory and motor cues. *Cognition*, 204, 104409.
- Rabini, G., Altobelli, E., & Pavani, F. (2019). Interactions between egocentric and allocentric spatial coding of sounds revealed by a multisensory learning paradigm. *Scientific reports*, 9(1), 1-12.
- Strelnikov, K., Rosito, M., & Barone, P. (2011). Effect of audiovisual training on monaural spatial hearing in horizontal plane. *PloS* *one*, 6(3), e18344.

Figure 4A legend appears to be reversed.

Corrected, thanks.

Line 439. To what extent are the head movements spontaneous in the training task? Are they not related to the task where the participant must visually guide the controller to the response location?

As the Reviewer says correctly, some head movements may be related to the fact that participants were facing the source in order to be able to make an accurate reaching movement to the designated visible speaker. However, there were also movement that were not directly related to hand reaching movement. As anticipated also above, the first head movement during the Spatial training started around 0.97 seconds after the beginning of the sound, but the hand response (irrespective of its correctness and registered as soon as participants reach the speaker using the controller) was provided around 3.78 seconds after the beginning of the sound. Moreover, the number of head-movement implemented during the Spatial training was 2.2 and they have been implemented during sound emission and not only close in time to the response.

Finally, we documented a head-rotation bias toward the plugged side during the sound emission irrespectively of the target positions. So, the movements of the head cannot be tied only to orienting it during the response because, on the contrary, the responses had a bias towards the space of the unplugged ear.

We have now integrated this aspect at page 21 and 27-28.

There were only 8 locations (4 azimuths and 2 elevations) in the testing phase. Did participants learn these locations over test trials? Localisation performance is different when from a limited set of alternatives than from an open set.

Yes, this is a possibility. We now discussed it as a possible explanation for the fact that we observed some improvements also after the Non-Spatial training (see pages 25-26). However, it is important to notice that during the head-pointing task, the speakers’ positions were entirely invisible. Furthermore, they changed their elevation with respect to training. Hence, participants did not have a-priori information about the distribution and numerosity of sound sources in space. Nevertheless, it is likely that participants increased their knowledge about possible sound positions during the task (e.g., they surely noticed that all sounds were in front space, and information that was also strengthened by vision of the speakers in the Spatial and Non-Spatial training tasks alike).

Line 506. Why were Phase and Training included as random effects?

Actually, we missed a part of the sentence in the original submission. Our apologies for this mistake. Now it reads: “To study the effects of our training protocols, we entered the CE values in monaural listening conditions into a LME model with PHASE (Pre or Post training), TRAINING (Spatial or Non-Spatial) as fixed effects, and PARTICIPANT and SESSION (intercepts) as random effects.”

Discussion:

Line 514. “that differ in terms of both stimuli and motor response.”
The stimuli for both training and test sessions was amplitude modulated white noise. The motor response for the test session was a head point to the target. The motor response for the spatial training was an arm reach, but it not clear to what extent participants also moved their heads. If they oriented toward the response location, then motor responses would be similar between training and test sessions, with the exception that training also required the arm reach. It is only if orienting head movements were not allowed during training that one can make a case for generalisation from arm reaching in training to head pointing in test.

Thanks for the observation. As you suggest, it could be that with the Spatial training based on reaching to sound we also implicitly trained head-pointing. However, as noted above, head-movements during training were not solely related to the reaching response. Actually, most head-movements preceded reaching, hence they were spontaneously implemented by participants. We added this aspect in the discussion on pages 27-28 and modified the sentence (old line 514). Also note that the effector used for the response was not the only difference between training and testing sound localization tasks. Both sound positions and sound type (2.5 Hz in the test and 2 or 3 Hz in the training) differed in the two tasks.

Line 601. An argument is made that changes in head movement may be a form of cognitive unloading due to the perceptually complex nature of the task. The rationale for this possibility is not well articulated. Head movements resolve ambiguity, but it is not clear that represents cognitive unloading.

We agree with the reviewer. We cannot demonstrate that it is cognitive offloading. We have now rewritten this part of the discussion and removed reference to the notion to cognitive offloading which was speculative.

The English expression is generally good, but there are a number of places that would benefit from proof reading, for example plurals expressed as singular etc.

We have carefully re-read the manuscript in the attempt to improve our use of English.

**Reviewer #2**

The aim of this study about adaptation to altered auditory cues is at least three-fold. It addresses whether a spatial auditory training procedure requires explicit or implicit processing of sound localization to be efficient; whether the training procedure resting on 13 specific sound positions (13 different azimuths) has also an impact on non-trained positions of the auditory space (4 different azimuths and 2 different elevations); and whether the spatial training based on reaching to sound is also efficient on localization performances through head orienting to sound.
This is a very nice study, carefully conducted, clearly written and presented.
I have only minor comments, that might help to make the message clearer.

Page 1, Author Note
Correct the typo in “Data can be retrieved from [osf.io/dt76.](http://osf.io/dt76.)” Since it is [osf.io/dt76h](http://osf.io/dt76h) as indicated later in the text.

Corrected, thank you.

Abstract:
“When the auditory cues change, sound localization becomes difficult and uncertain.”
This sentence is not easy to understand. Add “when auditory cues change, because of…or…., sound localization becomes ….”. Otherwise we do not grasp the meaning, since auditory cues change constantly.

We thank the reviewer. We have modified the sentences.

Highlights:
It would be nice to modify the last one to add how the findings can be applied to rehabilitation protocols.

We thank the reviewer. We have modified the last highlight (lines 46).

In the section “Procedure and stimuli”, please indicate the approximate duration of each of the different phases of the protocol. I could find it only for the AV cueing task.

We have added this information (pages 10 and 13).

Line 297 and 304, you mention that trials were removed from the analysis in the head pointing tasks and in the AV cueing task (incorrect responses). Please indicate for each task how many trials have been removed.

We have added this information (pages 14).

FIGURE 1: modify illustration C for more clarity. There is an arrow only on the right towards the non spatial training. Make sure that the illustration of the setup shared by the two types of training is in the middle of the figure.

We apologize to the reviewer. The arrow was lost. We have now loaded the correct Figure and coloured it to improve its clarity.

FIGURE 2: B and C. the absolute and signed errors are represented as a function of sound position in azimuth, but it is not straightforward to understand since the indicated azimuths on the x axis are not those that have been tested but only theoretical points (same for figure 3 and 4). Please also explain that the two tested elevations are cumulated for the analysis, taking into account azimuth only.

We have now added this information and explained the sound positions in the graphs.

Line 388 “The interval between the two testing sessions was effective in inducing a partial wash-out of training effects.”
Could you please discuss further this “partial” wash-out? How could you explain that the group with non-spatial training in the first session remained stable while the group with spatial training had a higher mean absolute error in the pre-test of session 2? Would it be relevant to do at the beginning of each session a binaural baseline?

We have further discussed and clarified the effects of the wash-out phase in the Discussion (pages 25-26), adding also new analyses to the manuscript (in footnote 2).

As to the possibility of introducing a second binaural baseline, we agree with the Reviewer that it would have been an additional piece of information for our conclusions. The current experiment and the corresponding Ethical approval were however related to a broader project involving a clinical population (people with deafness using cochlear implants). Hence, with the aim in mind to keep testing time to the very minimum we eventually opted for removing this additional phase in the study.

Line 411 “Localization errors in elevation remained unchanged, irrespective of training type.”
This finding is not discussed further, while it might be interesting. Might this difference be linked to the different mechanisms involved in the processing of binaural difference cues and in the processing of spectral cues? Monaural plugging alters dramatically binaural difference cues, but leaves unchanged monaural spectral cues on the remaining ear. Several findings suggest that those cues have an independent role in auditory localization (see for example Sarlat et al 2006). Alternatively, you might of course consider that the difference in elevation (10°) is so small that the absolute error exceeds the size of the difference between the two positions.

We further discussed this aspect in the Discussion (page 26).

Table 1: it would be nice to see somewhere the data for all the 8 stimuli instead of means of the 8 positions. Maybe it could be added in supplementary data?

We have now added another Table S2 in the supplementary data.

Line 507 Cueing effect: no main effects or interactions emerged. But on figure 7, and in its legend, an effect is marked. Please explain this effect in the text and comment on it. Indeed, on line 521 you state that the AV cueing task remained unaffected, which does not seem to be the case for the right targets in the post phase of the spatial training.

We apologize to the reviewer. A typo remained. Figure 7 has nothing to do with this task. We have now modified in the text.

Line 568 “in our experimental training there was no time restriction on sound duration, and participants experienced the acoustic inputs if they preferred (approximately 4 seconds).”

Do you know how long the participants took to answer (hence to listen to the stimuli) and could you analyse that?

We agree with the reviewer that this is a debatable component and a potential limitation of our study. We have now added information (see page 17 - Results and footnote 1 - Discussion).

In relation to your remark about the sound duration in line 566 "because the sounds lasted only 1.6 seconds" => 1.6 sec is quite a long duration. Goossens and Van Opstal (1999) observed heading with stimuli lasting only 500 ms.

Yes, the auditor is right. We intended, compared to our tasks, that it was 3 seconds for the test and “no time restriction” for the training. We rewrote this part (page 25).

Line 574-579 “First, reaching to sound could enhance the spatial coding of sound source location because results from the coordination of different effectors (eyes, head, hand) into a common reference frame (25,45). Second, reaching could help directing attention toward the position occupied by the sound source, making the task more engaging (fundamental to foster relearning, see Dehaene, 2020)(46). Third, reaching to sound could foster active listening through head exploration, as suggested by the documented shared reference frame between reaching motor command and head-orienting (47)."
I do not grasp the difference between the first and third proposition, especially in reference to the work of Boyer et al 2013 (ref 47). Please clarify.

We agree with the reviewer. We have corrected the reference and clarified (page 24).

In Supplementary material: The CE is now called “spatial advantage”, a notion that has not been introduced before.

We have now modified the name.
