# Supplementary material for: Adapting to altered auditory cues: Generalization from manual reaching to head pointing
Source: PLoS One. 2022 Apr 14;17(4):e0263509. doi: 10.1371/journal.pone.0263509 (PMC9009652; doi:10.1371/journal.pone.0263509)
Supplement: Attachment — Submitted filename: Response_01.2022.docx [file pone.0263509.s005.docx]

Reviewer #1: The revised manuscript is much improved. I have just a few minor comments and corrections.

The pictorial legend in Figure 4a remains reversed. Spatial – non spatial training should be open symbols.

We have now corrected the legend.

Line 421. I could not find Figure S2 in the Supplementary materials. Should it be Table S2?

Yes, we referred to Table S2.

Table S1. There was a significant interaction between Testing Phase and Azimuth for absolute errors. Does this mean that both spatial and non-spatial training were effective in reducing error on the plugged side?

Yes, as reported by the Reviewer, participants improved after both training (main effect of Phase, see also lines 401-403 in the manuscript). We now refer to this interaction also in the main text (lines 403-404). Crucially this improvement is more effective on the plugged side (as we can observe also in Figure 4B).

Table 1. Non-spatial training was also effective in reducing errors, albeit to a lesser extent than spatial training. This is addressed in the paragraph starting line 601, but it would be useful to include this in the Results.

In the Results, we wrote “We found a main effect of PHASE, caused by performance improvements after both training type”. As specified above we now report also the interaction between PHASE and AZIMUTH. These sentences describe indeed that both training determined an improvement. However, “the 2-way interaction between phase and training also reached significance […] After training, errors decreased more substantially after the Spatial compared to the Non-Spatial training”. This sentence describes that the. effects of Non-Spatial training were less pronounced (see lines 401-414).

Table S2 columns headers are -10 and 10. Should they be -15 and 5 given the offset between the controller and the speaker?

Yes, the Reviewer is right. We corrected this information.

Line 602. Training was not effective in improving vertical localisation. It could be the case that the reduced range of elevation (20 degrees) made improvements difficult to see, compared to the larger range of azimuths. Monaural cues to elevation are likely to be quite similar over a small range.

We added this remark (line 619-621).

Line 670. “overall” should read “overlap”

Corrected.

As suggested by the authors in the response to reviewers, it would be useful to include the data in the figures as well as the models to improve transparency of the results.

We modified Figure 2 and 4 to include the data.

There remain a few errors of English expression, primarily in the use of singular/plural.

We have now reread and corrected the manuscript.
